# Disseminated tuberculosis presenting with finger swelling in a patient with tuberculous osteomyelitis: a case report

**DOI:** 10.1186/1476-0711-4-18

**Published:** 2005-11-03

**Authors:** Shradha Agarwal, Daniel Caplivski, Edward J Bottone

**Affiliations:** 1 Mount Sinai Medical Center, One Gustave L. Levy Place, New York, New York, 10029. USA

## Abstract

**Background:**

Extrapulmonary manifestations of tuberculosis have become increasingly important in the era of HIV/AIDS.

**Case presentation:**

We describe a case of tuberculosis (TB) dactylitis in a patient with AIDS who originated from the Ivory Coast. The diagnosis was established by direct visualization of acid-fast bacilli on joint fluid and bone biopsy of the proximal phalanx. Imaging of the chest revealed multiple bilateral nodules. Confirmation of the diagnosis was made by isolation of *Mycobacterium tuberculosis *from sputum and bone cultures.

**Conclusion:**

Tuberculosis should be considered in patients with unusual soft tissue or skeletal lesions, especially when an immunosuppressive condition is present. Ziehl-Neelsen staining and culture of tissue obtained via surgical biopsy offer the most direct approach to diagnosis.

## Background

Tuberculous involvement of the metacarpals and phalanges is a rare presentation of extrapulmonary TB [1,2]. The spine is the most frequent site of skeletal involvement; occurring in 1 to 3% of patients with extrapulmonary TB [3]. The diagnosis is often delayed because osseous tuberculosis is a paucibacillary lesion and smears are often negative. We describe a patient with HIV infection from the Ivory Coast with finger swelling as the initial presentation of disseminated tuberculosis, including tuberculous osteomyelitis of the left phalanges. We review the incidence, diagnosis, and management of TB of the metacarpals and phalanges.

## Case Presentation

**Figure 1:**
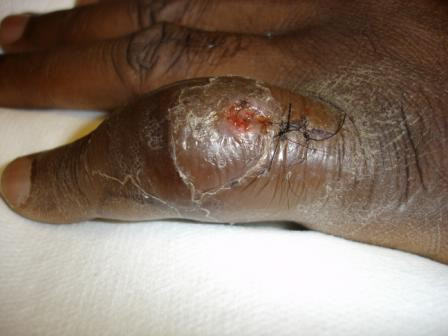
Left fifth digit with marked swelling. Photo taken after biopsy.

**Figure 2:**
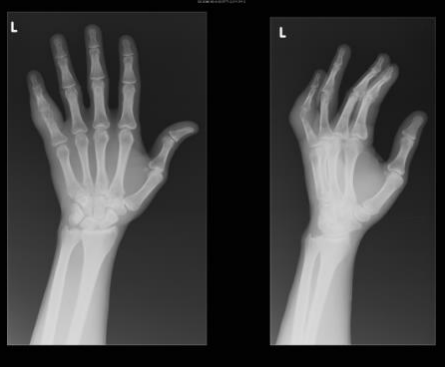
Plain radiograph of left hand demonstrated soft tissue swelling of the fifth digit, joint space narrowing with mottled lucency of the proximal phalanx, and cystic degenerative changes.

**Figure 3:**
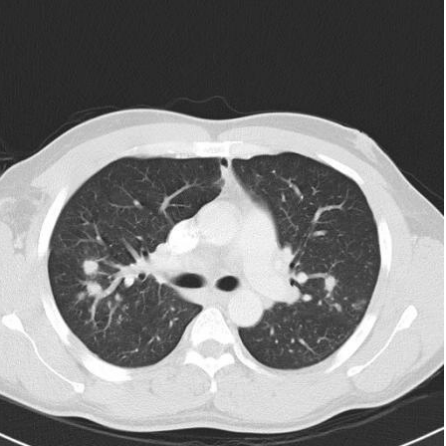
Computed Tomography Scan of the chest showed mediastinal lymphadenopathy and multiple bilateral pulmonary nodules.

A 46 year-old man with HIV/AIDS (CD4 T-cell 115 × 10^6^/l) presented with two weeks of left 5^th ^digit pain and swelling. He denied any history of recent trauma to the hand, fever, weight loss, or other systemic symptoms, but did note an occasional dry cough. He had traveled repeatedly between his native country Ivory Coast, West Africa and the United States. He was not taking antiretroviral medications at the time of presentation. On physical exam his fifth digit was swollen and erythematous at the proximal intraphalangeal joint [figure 1]. His laboratory values on admission were as follows: white blood cell count 5000 cells/mm^3^, hemoglobin 11.6 grams/dl, hematocrit 33.9%, platelets 280,000 platelets/mm^3^, eosinophils 9.0%, and erythrocyte sedimentation rate (ESR) of 60 mm/hour. Left hand radiography was significant for soft tissue swelling over the left finger with joint space narrowing and cortical lucencies with cystic degenerative changes in the proximal phalanx [figure 2]. Admission chest radiography demonstrated right hilar lymph node enlargement with multiple scattered nodules and a resolving right lower lobe infiltrate. Computed tomography scan of the chest revealed multiple pulmonary nodules [figure 3], necrotic lymph nodes, and splenomegaly. The patient underwent incision, drainage, and biopsy of the affected finger. Operative findings were significant for purulent, mottled, soft, yellow bone.

**Figure 4:**
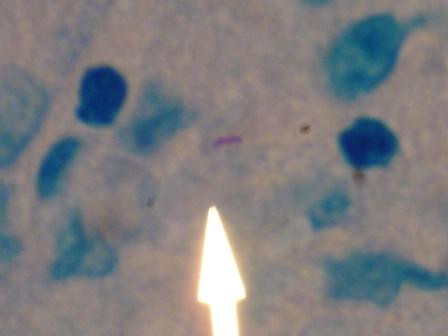
Biopsy of synovial tissue revealed rare slightly curved acid fast bacilli (Ziehl-Neelsen stain; original magnification ×1000).

**Figure 5:**
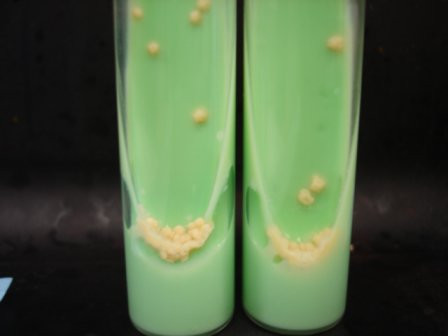
Culture on Lowenstein-Jensen medium revealed typical dry, heaped-up yellow to buff-colored colonies of *Mycobacterium tuberculosis*.

**Figure 6:**
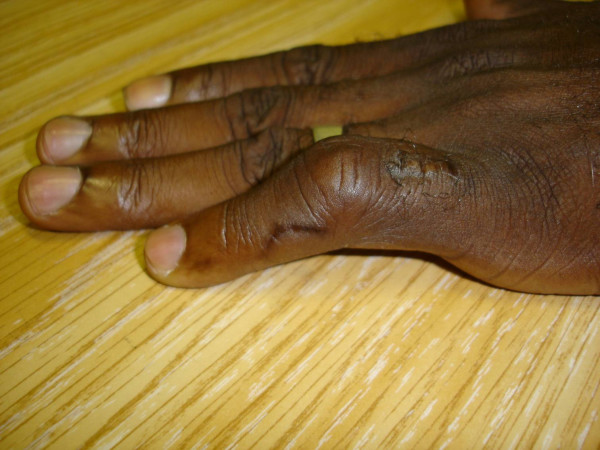
Follow-up photo of fifth digit 6 weeks after initiation of anti-tuberculous therapy.

Rare acid-fast bacilli were demonstrated on biopsy of the both phalanx and synovial fluid samples [figure 4]. We used the Gen-PROBE^® ^Amplified *Mycobacterium tuberculosis *Direct Test which employs a transcription-mediated amplification and hybridization protection assay to qualitatively detect *Mycobacterium tuberculosis *complex ribosomal RNA (rRNA). Several weeks later cultures of all surgical material grew *Mycobacterium tuberculosis *[figure 5]. Multiple Ziehl-Neelsen stains of induced sputum samples were negative for acid-fast bacilli, but all specimens sent for sputum culture grew *Mycobacterium tuberculosis*. Antituberculous treatment was initiated with rifampin, isoniazid, pyrizinamide, and ethambutol prior to culture results. After 12 weeks of treatment, marked improvement in the finger lesion was noted [figure 6].

## Discussion

Tuberculous involvement of the metacarpals and phalanges is a rare presentation of extrapulmonary TB [1,2]. Although rare in adults, 85% of patients with TB dactylitis are younger than 6 years of age and bones of the hands (specifically the proximal phalanx of the index and middle fingers) are more frequently affected compared to bones of the feet [4]. Osteoarticular involvement occurs in 1 to 3% of patients with extrapulmonary TB [3], the spine representing 50% of these lesions [5]. Only 1/3 of patients with tuberculosis of the bone are diagnosed with concomitant active pulmonary disease [6]. Extrapulmonary tuberculosis presents more frequently in patients with CD4 counts less than or equal to 100 × 10^6^/L [7]. The diagnosis is often delayed because osseous tuberculosis is a paucibacillary lesion, and smears are often negative. Localized pain, fever, and weight loss are described in several case reports of musculoskeletal tuberculosis [8,9]. Our patient presented with a painful, swollen finger as the initial manifestation of disseminated tuberculosis.

The radiographic features of osseous tuberculosis (sclerosis and osteolytic lesions) are present in conditions such as inflammatory arthritis, pyogenic osteomyelitis, Brodie's abscess, Kaposi's sarcoma, and other malignancies. Other findings on plain radiographs include osteopenia, soft-tissue swelling with minimal periosteal reaction, narrowing of the joint space, cysts in bone adjacent to joints, and subchondral erosions [10,11]. The non-specific nature of these radiographic findings can often delay the diagnosis [12].

The gold standard for the diagnosis of osseous tuberculosis is culture of *Mycobacterium tuberculosis *from bone tissue. Positive Ziehl-Neelsen staining for acid-fast bacilli requires at least 10^4 ^acid-fast bacilli per milliliter of specimen and does not differentiate between tuberculous and non-tuberculous mycobacteria [13,14]. The advent of DNA detection via PCR may increase sensitivity and allow for the exclusion of non-tuberculous mycobacteria (such as *M. marinum*) that also cause soft tissue infections.

Current recommendations for the treatment of osseous tuberculosis include a 2-month initial phase of isoniazid, rifampin, pyrazinamide, and ethambutol followed by a 6- to 12-month regimen of isoniazid and rifampin [15]. There are few studies that define the optimal duration of treatment of skeletal tuberculosis involving the hand. Some experts favor a prolonged course of therapy to optimize post-treatment function. Others argue that the paucibacillary nature of the lesion make a 6-month treatment course appropriate. Subasi, et al. [1] describe the treatment and outcome of a series of 7 patients with tuberculosis of the metacarpals and phalanges. These patients were treated with a 4-drug regimen for 2 months, followed by a 2-drug regimen for 10 months. Within 6 weeks, patients demonstrated clinical signs of healing and improvement in joint motion. Upon final evaluation (mean follow-up 30 months), all patients had healed lesions without recurrence and none required arthrodesis.

Finger swelling is a rare presenting sign of disseminated tuberculosis. This case illustrates the importance of a high index of suspicion when evaluating a patient with an unusual destructive bony lesion. Early biopsy and appropriate microbiologic testing can avoid diagnostic delay.
